# Transcriptome data of peripheral white blood cells from beef heifers collected at the time of artificial insemination

**DOI:** 10.1016/j.dib.2018.03.062

**Published:** 2018-03-21

**Authors:** Sarah E. Dickinson, Fernando H. Biase

**Affiliations:** Department of Animal Sciences, Auburn University, 599 Devall Dr, Auburn, AL 36839, United States

**Keywords:** AI, artificial insemination, FTAI, fixed time artificial insemination, mRNA, messenger ribonucleic acid, NB, natural-bred, PWBC, peripheral white blood cells, RNA, ribonucleic acid, Biomarkers, Infertility, Pregnancy

## Abstract

The reproductive performance of heifers within their first breeding season influences the success of beef cattle operations. Therefore, a means to identify infertile and late breeding heifers before the start of the breeding season holds great promise for the future of the beef industry. Pubertal beef heifers were subjected to estrous synchronization and fixed time artificial insemination (FTAI). We collected peripheral blood from the heifers at the time of artificial insemination (AI) and generated RNA sequencing data to characterize the transcriptome of peripheral white blood cells (PWBC). Following insemination, heifers were exposed to natural service for a defined breeding season, and pregnancy was evaluated to classify heifers into one of three groups: AI-pregnant, natural-bred (NB) pregnant, and non-pregnant. The raw transcriptome data of PWBC is available on the NCBI GEO repository (GSE103628) where the reader can also find raw read counts and normalized gene expression data. The normalized data on transcript coverage can be visualized as a genome browser at HeiferFertilityRNAseq.org.

**Specifications table**

**Table:** 

Subject area	*Biology, Animal Sciences*
More specific subject area	*Reproductive Biology, Genomics*
Type of data	*mRNA sequencing data*
How data was acquired	*Next Generation Sequencing*
Data format	*High throughput sequencing short reads*
Experimental factors	*Samples were collected from heifers of three fertility classifications from two different locations.*
Experimental features	*Blood was collected from crossbred, pubertal beef heifers (n=23) at the time of artificial insemination(AI). Heifers were then exposed to natural service for a defined breeding season. Pregnancy was evaluated, and heifers were categorized as AI-pregnant, NB-pregnant, or not pregnant. RNA was extracted from PWBC of each heifer and used as the template for cDNA libraries that were sequenced on an Illumina HiSeq. 2500.*
Data source location	*Auburn, Alabama*
Data accessibility	*Raw data is in the public NCBI GEO repository (GSE103628,*https://www.ncbi.nlm.nih.gov/geo/query/acc.cgi?acc=GSE103628*).* N*ormalized data on transcript coverage can be visualized as a genome browser at HeiferFertilityRNAseq.org (Fig. 1).*

**Value of the data**

• The data can be used to expand knowledge of transcriptome characteristics of beef heifers with varying fertility potential.
• The data can be used to increase understanding of physiological components of fertility.
• The data benchmark the importance of investigating mRNA abundance of specific genes for the identification of biomarkers for infertility or subfertility in beef heifers.

## 1 Data

Data presented in this article correspond to high throughput RNA-sequencing of PWBC collected at AI from heifers of three distinct pregnancy outcomes: AI-pregnant, NB-pregnant, or non-pregnant following a defined first breeding season. The interpretation of these data is presented in the following research article: Dickinson et al. 2018. Transcriptome profiles in peripheral white blood cells at the time of artificial insemination discriminate beef heifers with different fertility potential [1].

## 2 Experimental design, materials and methods

### 2.1 Experimental design

We generated RNA-sequencing data from 23 heifers from two Auburn University Alabama Agricultural Experimental Stations. Sequencing data represents PWBC mRNA from blood collected at the time of AI from heifers that were classified as AI-pregnant, NB-pregnant, or non-pregnant after their first breeding season.

### 2.2 Materials and methods

#### 2.2.1 Animal handling

All procedures for animal handling were performed in accordance with the protocols approved by Institutional Animal and Care and Use Committee in Auburn University. All animal experiments were carried out in accordance with the National Institutes of Health guide for care and use of Laboratory Animals. Sixty pubertal, crossbred heifers (Angus × Simmental; 13.5 months of age) were subjected to estrous synchronization and fixed time AI with a 7-day CO-Synch protocol. Fourteen days after AI, the heifers were exposed to natural breeding for a defined breeding season of 86 days (station A) or 42 days (station B). An experienced veterinarian performed pregnancy evaluation by transrectal palpation on day 62 and 125 post insemination at station A, and on day 95 post insemination at station B. Presence or absence of a conceptus, alongside morphological features indicating fetal age were recorded, and heifers were classified as pregnant to AI, pregnant to natural service, or non-pregnant.

#### 2.2.2 Selection of heifers for RNA sequencing

We selected heifers from each experimental station for transcriptome high throughput sequencing. From station A, we characterized the transcriptome of PWBC from AI-pregnant (N = 6) and NB-pregnant (N = 5) heifers. From station B, we determined PWBC transcriptomes of AI-pregnant (N = 6) and non-pregnant (N=6) individuals.

#### 2.2.3 Blood collection and processing

At the time of AI, 10 ml of blood was drawn from the jugular vein into vacutainers containing 18 mg K2 EDTA (Becton, Dickinson and Company, Franklin, NJ). The tubes were inverted for 8–10 times and placed in ice for transport to the laboratory. The tubes were centrifuged for 10 minutes at 2000g at 4 °C. The buffy coat was removed and placed into 14 ml of red blood cell lysis solution (0.15 M ammonium chloride, 10 mM potassium bicarbonate, 0.1 mM EDTA, Cold Spring Harbor Protocols) for 10 minutes at room temperature (24–25 °C). The solution was discarded and the pellet was re-suspended in 200 µl of RNAlater® (Lifetechnologies™, Carlsbad, CA). The PWBCs were then stored at −80 °C prior to RNA extraction.

#### 2.2.4 RNA extraction, library preparation, and high throughput sequencing

Total RNA was isolated from PWBCs of 23 heifers using TRIzol™ reagent (Invitrogen, Carlsbad, CA) following the protocol recommended by the manufacturer. RNA yield was quantified using the Qubit™ RNA Broad Range Assay Kit (Eurogene, OR) on a Qubit® Fluorometer, and integrity was assessed on Agilent 2100 Bioanalyzer (Agilent, Santa Clara, CA) using an Agilent RNA 6000 Nano kit (Agilent, Santa Clara, CA). Libraries were prepared with the TruSeq Stranded mRNA Library Prep kit (Illumina, Inc., San Diego, CA). Libraries were quantified with Qubit™ dsDNA High Sensitivity Assay Kit (Eurogene, OR) and quality was evaluated using the Agilent 2100 Bioanalyzer High Sensitivity DNA chip (Agilent, Santa Clara, CA) on an Agilent 2100 Bioanalyzer (Agilent, Santa Clara, CA). Libraries were sequenced in a HiSeq. 2500 system at the Genomic Services Laboratory at HudsonAlpha, Huntsvile, AL to generate 125 nucleotide long pair-end reads.
